# Younger North Americans are exposed to more radon gas due to occupancy biases within the residential built environment

**DOI:** 10.1038/s41598-021-86096-3

**Published:** 2021-03-24

**Authors:** Justin A. Simms, Dustin D. Pearson, Natasha L. Cholowsky, Jesse L. Irvine, Markus E. Nielsen, Weston R. Jacques, Joshua M. Taron, Cheryl E. Peters, Linda E. Carlson, Aaron A. Goodarzi

**Affiliations:** 1 Faculty of Medicine, University of Saskatchewan, Saskatoon, SK Canada; 2 Robson DNA Science Centre, Departments of Biochemistry and Molecular Biology and Oncology, Charbonneau Cancer Institute, Cumming School of Medicine, University of Calgary, Calgary, AB Canada; 3 School of Architecture and Landscape Planning, University of Calgary, Calgary, AB Canada; 4 Departments of Cancer Epidemiology and Prevention Research and Community Health Sciences, Charbonneau Cancer Institute, Cumming School of Medicine, University of Calgary, Calgary, AB Canada; 5 Division of Psychosocial Oncology, Department of Oncology Charbonneau Cancer Institute, Cumming School of Medicine, University of Calgary, Calgary, AB Canada

**Keywords:** Cancer epidemiology, Cancer prevention, Risk factors, Lung cancer

## Abstract

Residential buildings can concentrate radioactive radon gas, exposing occupants to particle radiation that increases lung cancer risk. This has worsened over time in North America, with newer residences containing greater radon. Using data from 18,971 Canadian households, we calculated annual particle radiation dose rates due to long term residential radon exposure, and examined this as a function of occupant demographics. The current particle radiation dose rate to lungs from residential radon in Canada is 4.08 mSv/y from 108.2 Bq/m^3^, with 23.4% receiving 100–2655 mSv doses that are known to elevate human cancer risk. Notably, residences built in the twenty-first century are occupied by significantly younger people experiencing greater radiation dose rates from radon (mean age of 46 at 5.01 mSv/y), relative to older groups more likely to occupy twentieth century-built properties (mean age of 53 at 3.45–4.22 mSv/y). Newer, higher radon-containing properties are also more likely to have minors, pregnant women and an overall higher number of occupants living there full time. As younger age-of-exposure to radon equates to greater lifetime lung cancer risk, these data reveal a worst case scenario of exposure bias. This is of concern as, if it continues, it forecasts serious future increases in radon-induced lung cancer in younger people.

## Introduction

Lung cancer causes ~ 40% of North American cancer-related deaths, and lung cancer in never-smokers is the 7th leading cause of all cancer-related mortality globally^1,2^. Unlike tobacco-induced lung cancer, incidence of lung cancer in North American never-smokers is still increasing, encompassing 1 in 5 lung cancers^3–6^. At the population level, radon gas inhalation is the 2nd leading cause of lung cancer^4,7–13^ and is responsible for tens of thousands of new cases and related deaths per year^7,10–16^. Radon-222 (^222^Rn) gas is radioactive and is generated naturally in soil across the world, although it normally dilutes to non-hazardous levels in outside air once it reaches the earth’s surface^14^. However, 20th-21st–century buildings can capture, contain and concentrate radon to unnatural and increasingly hazardous levels, creating a human-made radiation issue within our built environment. Unfortunately, this is worsening in twenty-first century-built North American residential properties, which contain substantially greater radon gas levels relative to those constructed during the twentieth century (and earlier) for not yet entirely clear reasons relating to evolving build practices^12,13^. Canadians are at particularly great risk of excessive radon gas inhalation within their residential environment, with exposure across the Prairies found recently to be amongst the highest in the world. Indeed, it is estimated that long term residential radon inhalation is responsible for causing > 1 new lung cancer *per day* in some hard-hit Canadian provinces^2,7–9,11^.

The *International Agency for Research on Cancer* defines radon as a category 1 carcinogen. This is because ^222^Rn emits alpha particle ionizing radiation that damages DNA in a way nearly impossible to heal without genetic errors that drive cancer formation^14–16^. Radiation susceptibility in terms of cancer risk varies across populations, with approximately 1 in 30 North American adults displaying genetically-mediated radiation sensitivity^15,17–25^. Distinctly elevated risks from radon exposure are also observed in women and children^11–13,26–29^. Lifetime relative risk of lung cancer from radon is inversely proportionate with age, with the youngest being the most at risk due to innate pediatric radiosensitivity, faster breathing rates, lower body mass and, most potential years of life lost at time of exposure^15,16,26^. Thus, when considering cancer risks from radiation, it is essential to understand age at time of exposure.

Alpha particle radiation is measured in Becquerels (Bq) per m^3^, equating to one radioactive decay event per second per cubic metre of air. A 16% increase in relative lifetime risk of lung cancer occurs per ≥ 100 Bq/m^3^ long term radon inhalation, and has an even greater synergistic increase in patients who also smoke tobacco^10,11,14,15,30^. Indeed, radon exposure is very important to consider the case of light smokers, whose tobacco-related cancer risk might be low, but if combined with radon can become as high as a heavy smoker, as the differing types of DNA damage elicited by tobacco carcinogens (nitrosamines, benzene, aldehydes and polyaromatic hydrocarbons) combine with the effects of alpha particle radiation-induced ionization to increase genomic instability. In biology and medicine, radiation exposure is measured in Sieverts (Sv), representing absorbed energy per unit of mass and weighted to account for relative biological effectiveness. Bq/m^3^ of radon can be converted to mSv radiation exposure using a formula standardized by the *International Commission for Radiological Protection* (ICRP). Our study objective was to define mSv/y lung radiation dose exposures attributable to radon inhalation from the Canadian residential environment, and examine this as a function of building construction year and occupant ages.

## Results

### Residential building radon exposure and human occupancy data

**Figure 1:**
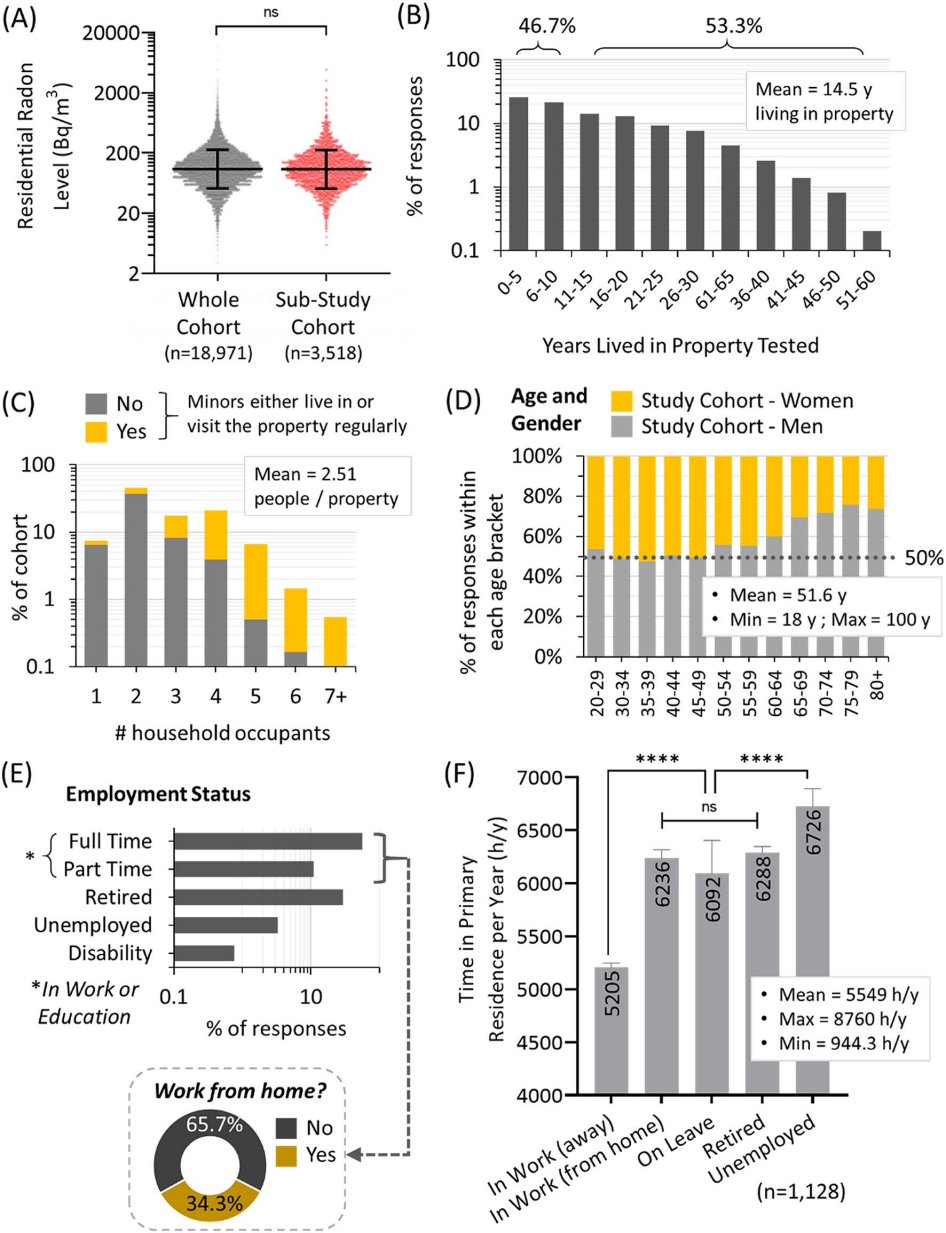
Residential occupancy status, age and employment status of the radon-tested cohort. (**A**) The measured long term residential radon level for the entire Canadian study cohort (grey: n = 18,971) was compared to the sub-study (red: n = 3,518), i.e. participants who also provided demographic data. The radon test outcomes for the sub-study reflected the entire cohort, with no significant difference in the geometric mean radon (black lines ± geometric standard deviation) or data distribution between sets. All remaining data refers to the sub-study cohort unless otherwise indicated. (**B**) The amount of time each participant indicated they had lived in the radon-tested property at the time of testing. (**C**) The total number of occupants living full time in the radon-tested property, with yellow indicating properties where there are pregnant occupants or minors (ages 0–17) living or visiting regularly, and grey sections indicating homes where all occupants are 18 or more years of age. (**D**) Reported gender balance by age group of the cohort. (**E**) The reported employment status of the cohort. The inset pie chart shows the percentage of those in full or part time work who indicate working from home either part or all of the time. (**F**) The geometric mean of the amount of time each participant reported spending per year in the primary residence, as a function of reported occupation status, showing 2-way ANOVA analysis of indicated pairwise comparisons. ****p < 0.0001.

The total residential radon dosimetry dataset includes outcomes of 18,971 long term alpha track radon tests conducted between 2015–2020 using a controlled citizen science approach outlined in^12,13,31^. Canadian residences contained a geometric mean radon level of 108.2 Bq/m^3^ (CI_95%_ [107.1, 109.4]) with 1 in 6 being ≥ 200 Bq/m^3^, a common administrative exposure limit in Canadian and European jurisdictions. The median test duration was 121.7 days, with a majority of tests being conducted between September to June and without significant seasonal variation as per our previous work^12^. Of these radon-tested properties, 3,518 participants provided additional demographic and building occupancy data. There was no statistical difference (*p* > 0.05) in geometric mean radon level or data distribution between the main cohort and the sub-study cohort (Fig. 1A). The average time that participants reported living full time in the radon-tested property at the time of radon testing was 14.5 years (y), with 53.3% living there for ≥ 10 y (up to a maximum of 60 y) and 46.7% reporting living there < 10 y (Fig. 1B). The geometric mean number of occupants per property was 2.5, with those properties with 4 or more occupants being more likely to include minors (ages 0–17) (Fig. 1C).

The binary gender balance for the primary respondents was equal for those under 60, with a modest skew towards men for those 60 and older (Fig. 1D). The majority of participants report being in full (56.7%) or part (10.9%) time employment at the time of the survey; the remainder being retired (29.1%), unemployed (3.3%) or on disability leave (0.77%) (Fig. 1E). Of those in work, 34.3% reported doing so from home some or all of the time. Of the cohort providing demographic data, 1,128 participants also provided detailed occupancy data for their primary residence, which we then analyzed as a function of employment status (Fig. 1F). Those in work (or enrolled in formal education) spent an average of 5205 h/y at home, with those working-from-home, retired or on leave spending ~ 6200 h/y, and the unemployed spending 6726 h/y. Collectively, the entire cohort reported spending 5549 h/y inside their primary residence, which (allowing for time spent in other residences per year) fits well with data obtained from the Canadian and American *National Human Activity Pattern Study*^32^ that found the average adult spent 68.7% (6018 h/y) of life inside a residence.

### Residential radon radiation exposure dosimetry

**Figure 2:**
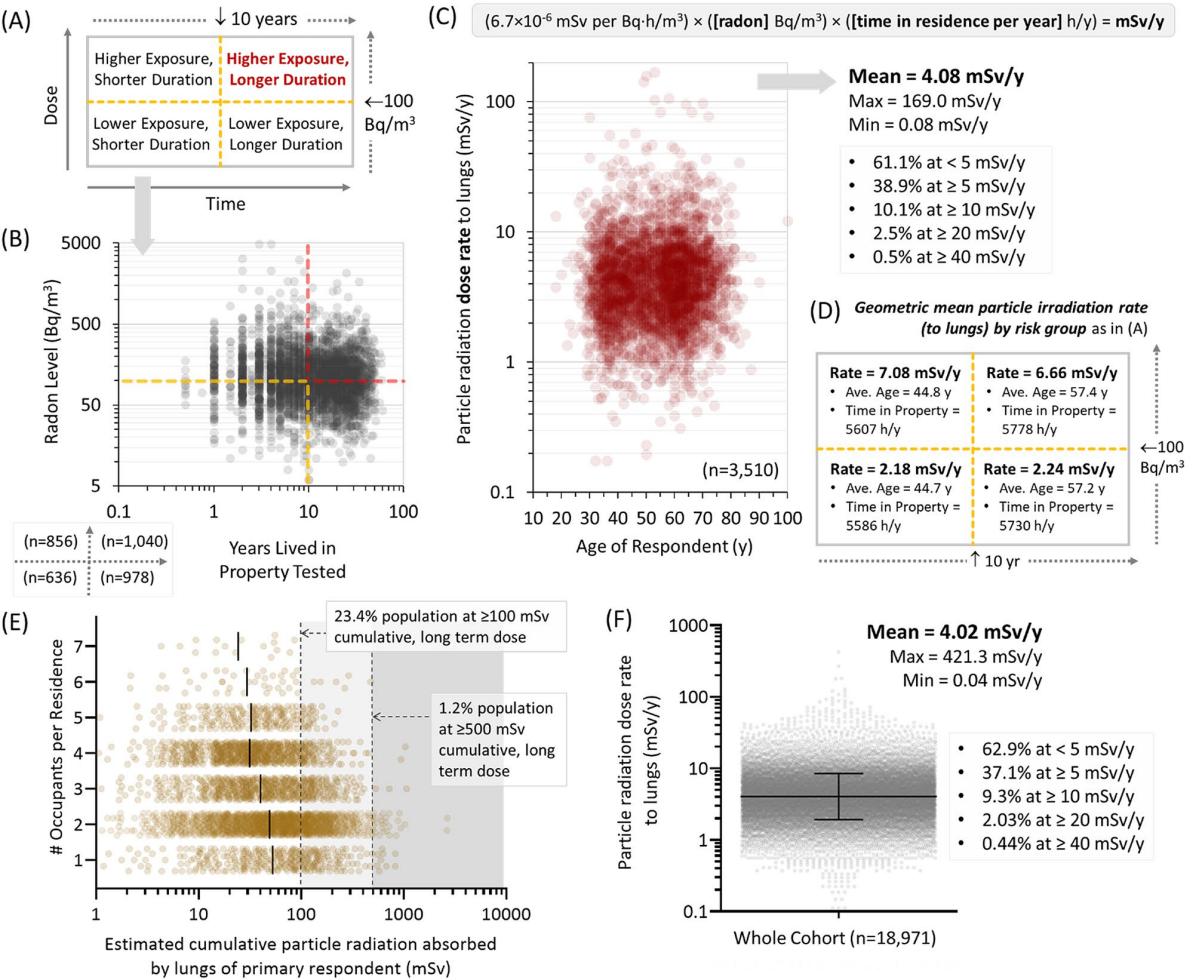
Yearly and cumulative particle radiation exposure from radon inhalation. (**A**). Rubric delineating residential radon exposure (high exposure =  ≥ 100 Bq/m^3^) as a function of the amount of time a participant reports living in that property (long exposure =  ≥ 10 y). (**B**) Residential radon exposure as a function of years lived in that environment, with red and yellow lines demarcating exposure brackets outlined in (**A**). (**C**) Using the indicated formula from ICRP (see methods), Bq/m^3^ radon levels were combined with exposure durations to derive mSv/y particle radiation dose rates, expressed as a function of participant age. The geometric mean dose rate and the percent of the cohort experiencing < 5, ≥ 5, ≥ 10, ≥ 20, or ≥ 40 mSv/y are indicated. (**D**) The particle radiation dose rate (mSv/y) was broken down by exposure brackets outlined in (**A**), additionally indicating the geometric mean of participant ages and the time they reported spending in their primary residence per year. (**E**) The estimated cumulative particle radiation exposure for all primary respondents (in mSv) was expressed as a function of the number of occupants for that specific property. (**F**) The particle radiation dose rate (mSv/y) for the larger cohort of radon tested, properties (n = 18,971) was estimated using the geometric mean amount of time each participant reported spending per year in the primary residence (5549 h/y). The geometric mean annual particle dose rate and the percent of the cohort experiencing < 5, ≥ 5, ≥ 10, ≥ 20, or ≥ 40 mSv/y are indicated.

To reflect the most common modes of residential radon exposure information available to the general public (i.e. radon test data from their current residence), we divided our data into four categories as in Fig. 2A. In short, 100 Bq/m^3^—where a statistically significant increase in relative lifetime risk of lung cancer occurs^14,33,34^—was used as a cut-off to define higher (≥ 100 Bq/m^3^) or lower (< 100 Bq/m^3^) radon exposure. A decade of exposure within the residence was used as a convenient cut-off to define longer (≥ 10 y) or shorter (< 10 y) duration, based on the rationale that the latency period between radon exposure to lung cancer formation is measured in decades^5,6,14^. Individual outcomes within our cohort split well between these four categories (Fig. 2B). We next converted Bq/m^3^ indoor air radon levels to particle radiation exposure rates (to adult lungs) in mSv/y using ICRP formula (see methods), and expressed this as a function of each respondents age at the time of surveying (Fig. 2C). To calculate mSv/y, we used the precise amount of time that 1,128 participants reported spending inside their primary residence in a given year or, for the rest (n = 2,390), an informed value based on individually reported employment status and the data obtained in Fig. 1F. The geometric mean annual alpha particle radiation dose rate to lungs from current Canadian residential radon exposure was determined to be 4.08 mSv/y (min 0.08 mSv/y; max 169.0 mSv/y). Of this population, more than 10% currently receive ≥ 10 mSv/y from radon in their residence. These radiation exposure rates were then analyzed by the groups defined in Fig. 2A. Those in higher exposure categories encompassed 54% of respondents experiencing 6.66–7.08 mSv/y, with all other categories being lower by threefold or more (Fig. 2D).

To gain a sense of cumulative particle radiation exposure over the amount of time that each individual spent within the radon-tested environment, we next multiplied dose rates by the amount of time each person reported living full time in that property, and expressed these values as a function of the number of occupants. For the purposes of modelling, we operated on the assumption that radon levels within each property did not fluctuate significantly from year to year over the long term. This is supported by our previous work that found no year-on-year variations in the collective radon test outcome for North American large populations^12^, and the fact that all participants in this cohort verified that they had not engaged in any type of radon mitigation of their primary residence at any time before surveying. Estimated, cumulative particle radiation doses were an average of 40–50 mSv, with 23.4% of the population receiving ≥ 100 mSv, and 1.2% receiving ≥ 500 mSv to the lungs over the long term from residential radon inhalation (Fig. 2E). To estimate mSv/y particle radiation dose rates for a much larger population, we used the average amount of time our cohort spent in their primary residence per year (5549 h/y), and our larger radon test dataset as in Fig. 1A. The geometric mean outcome of 4.02 mSv/y compared well with the outcomes of our sub-study cohort in Fig. 2C, with the overall maximum dose rate from this larger population being 421.3 mSv/y (Fig. 2F).

### Residential Radon exposure by age of property and occupant demographics

**Figure 3:**
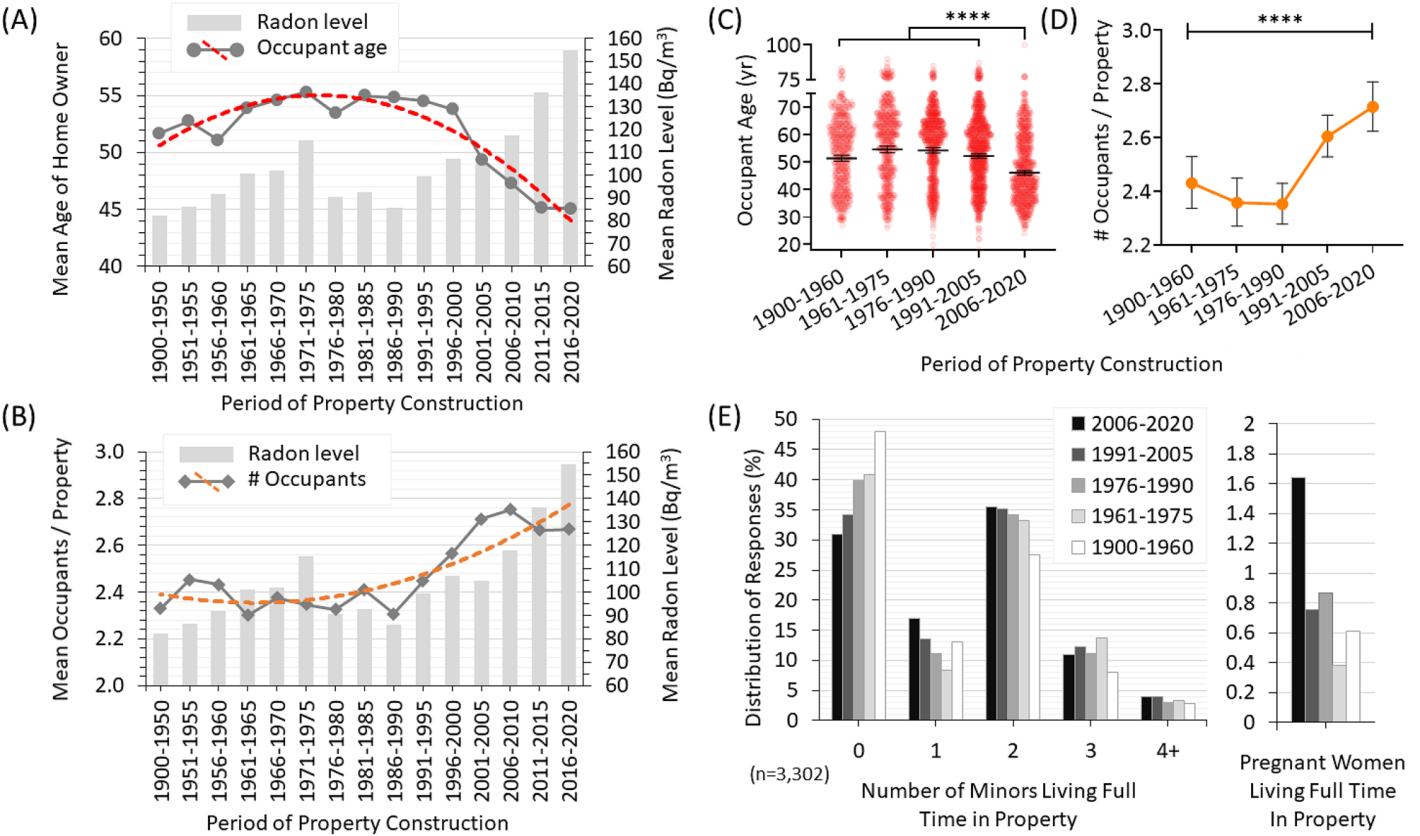
Canadian radon exposure as a function of residence age, occupant age, number of occupants and presence of minors and pregnant occupants. (**A**) Long term radon-test outcomes (grey bars, right y-axis) and geometric mean age of primary homeowner/renter (grey circles, line overlaid with red dashed polynomial trendline, left y-axis) for Canadian residences grouped into 5 year bracket (x-axis). (**B**) Long term radon-test outcomes (grey bars, right y-axis) and geometric mean age of the number of full time occupants (grey diamonds, line overlaid with orange dashed polynomial trendline, left y-axis) for Canadian residences grouped into 5 year bracket (x-axis). (**C**) Scatter plot of occupant age data from (**A**) by property construction period, showing 2-way ANOVA analysis of properties from 2020–2006 pairwise compared to all other period brackets. ****p < 0.0001. (**D**) Line graph of the geometric mean occupants per property (from **B**), showing 2-way ANOVA analysis of all comparisons. ****p < 0.0001. (**E**) The percent distribution of responses for reported number of minors (or pregnant women) living full time within the property, grouped by period of property construction.

We next sorted all participants based on the construction year of their specific residence, ranging from 1900 to 2020, and linked this to the average long term radon test outcomes (light grey bars, right y-axis) of those properties (Fig. 3A-B). As observed previously^12,13^, newer North American properties contained greater radon gas, with those constructed in the twenty-first century having the highest levels. We then analyzed these data as a function of the primary homeowner or renter’s reported age (dark grey line with dashed red polynomial trendline, left y-axis) (Fig. 3A), or the total number of occupants reported per residence (dark grey line with dashed orange polynomial trendline, left y-axis) (Fig. 3B). There was a clear trend wherein the newest properties had the highest radon and also the greatest number of occupants of the youngest overall ages.

All observations, coupled with geometric mean particle radiation dose rates, are summarized in Table 1. Construction periods were grouped into four 15 year brackets going back to 1960, with older properties (1900–1960) in a fifth bracket. People in properties built from 2006 to 2020 had the highest documented annual particle radiation exposure (to lung) dose rate, at 5.01 mSv/y, the most starkly contrasted to those in properties built from 1976 to 1990, who experience the substantially lower 3.60 mSv/y. While there was no statistically significant difference (*p* > 0.05) in the geometric mean age of occupants living in properties constructed from 1900 to 2005 (~ 53 ± 1.3 y), those in properties built within the past 15 y (2006–2020) were significantly (*p* < 0.0001) younger being a geometric mean age of 46.1 (CI_95%_ [45.3, 46.9]) (Table 1, Fig. 3C). Properties from this period were also home to a significantly (*p* < 0.0001) greater number of occupants relative to all other property build periods (Table 1, Fig. 3D). Finally, we examined how many minors (ages 0–17) or pregnant occupants were reported to live full time in each property by the primary respondent (homeowner / renter), as a function of year of construction (Fig. 3E). People in the newest properties were the most likely to be caring for a first and/or second child, were the least likely to have no children present at all, and reported more than double the number of full time occupants who were pregnant compared to all other property ages.

**Table 1:** Summary of North American radiation exposure from residential radon.

Period of construction	Radon Level (Bq/m3)					Radiation Dose (mSv/y)					# Occupants	Age of owner
	Geomean	Percentiles		Max	Min	Geomean	Percentiles		Max	Min	Geomean	Geomean
	[CI_95%_]	95% mSv/y	5% mSv/y			[CI_95%_]	95% mSv/y	5% mSv/y			[CI_95%_]	[CI_95%_]
1900–1960	**92.0** [86.9, 98.7]	345.0	31.0	1599	2	**3.45** [3.2, 3.7]	14.91	0.97	55.5	0.08	**2.50** [2.4, 2.5]	**51.4** [50.3, 52.6]
1961–1975	**109.3** [101.6, 113.9]	315.0	33.6	1666	6	**4.22** [3.9, 4.5]	12.95	1.30	77.4	0.18	**2.36** [2.3, 2.5]	**54.7** [53.5, 55.9]
1976–1990	**93.7** [88.5, 97.7]	307.5	28.0	1303	6	**3.60** [3.4, 3.8]	13.05	1.11	29.1	0.18	**2.35** [2.3, 2.5]	**54.4** [53.4, 55.3]
1991–2005	**107.8** [103.4, 112.3]	328.8	35.8	2145	8	**4.11** [3.9, 4.3]	12.66	1.28	106	0.39	**2.60** [2.5, 2.7]	**52.3** [51.5, 53.0]
2006–2020	**133.4** [127.2, 141.6]	438.9	42.0	4852	11	**5.01** [4.7, 5.3]	18.51	1.55	169	0.53	**2.71** [2.6, 2.8]	**46.1** [45.3, 46.9]

## Discussion

Understanding Bq/m^3^ property radon levels is important, but extrapolating long term absorbed alpha particle irradiation doses to the lungs is more informative for understanding biology and forecasting medical outcomes. This work finds that the doses and dose rates of particle radiation that North Americans are receiving from residential radon are within the ranges verified to increase cancer risk in humans and animal models. Our data also reveal a troubling, age-related bias in particle radiation exposure due to residential radon, where the youngest North Americans are more likely to reside within indoor air environments with the largest doses and dose rates. This younger population was also more likely to include pregnant women or groups caring for, what is reasonable to infer are, the youngest children. Given that younger people, women and children are the most prone to negative health effects from radon^11–13,26–29^, this is a worst case scenario in terms of lung cancer and radiation exposure risk. Similar to other lung carcinogens (e.g. tobacco, asbestos), the latency period between radon gas exposure and lung cancer onset is between one to three decades^5,6,14^. This means that unless action is taken soon to reduce historically high (and still rising^12^) North American residential radon exposure that unfavourably impacts the youngest age groups, within the next few decades this region is likely to face an unprecedented increase in new lung cancers among those who are in the ‘prime of life’.

These concerns are supported by multiple, large epidemiological pooling studies that confirm linear increases in relative lifetime risk of lung cancer at ≥ 100 Bq/m^3^ of repetitive, long term radon exposure^14,33,34^. Human cancer risk commences at a cumulative dose of 50–100 mSv prolonged exposure to ionizing radiation such as x-rays^35^, and at substantially lower doses for higher linear energy transfer ionizing radiation such as particles from radon^36^. Occupational safety guidelines from the *Canadian Nuclear Safety Commission* tolerate a maximum radiation exposure dose of 5 mSv per year, or 20 mSv spread over 5 years. Animal models demonstrate that 2500–5000 mSv total exposure at ~ 12.5 mSv/week particle radiation from long term radon gas inhalation produced lung cancers in 30–70% of exposed male rats^36^. It is important to understand that doses of radon inhaled by animals at lower dose rates were *more* lung cancer-causing compared to the same doses inhaled at higher dose rates (e.g. the 2500–5000 mSv total dose delivered at ~ 125 mSv/week of radon inhalation caused lung cancer in only 10–30% of rats). This makes sense from a radiobiological perspective, as higher and faster doses are more likely to kill exposed lung cells outright while, at lower dose rates, cells experience elevated genetic mutation but not death and are so able to progress into oncogenic transformation and cancer^15,16^. Worryingly, our work finds that North Americans are experiencing cancer-causing dose rates and cumulative exposures of particle radiation from residential radon, and that these are even higher for younger people who are more likely to live in newer properties (see Table 1).

The age-related home occupancy biases we observe are likely explained by data demonstrating that North American first time home-seekers (i.e. ages 24–44) have more limited financial resources that preclude buying or renting the typically more expensive residential properties in older and “more established” neighbourhoods, and/or are preferring newer properties with more modern design trends that are also smart-home ready and/or energy efficient^37–39^. This phenomenon is exemplified by the proliferation of suburban sprawl, ‘bedroom communities’ and ‘commuter towns’ across North America to meet the growing housing demands of younger people priced out major metropolitan city centres^39–41^. This is a rising public health issue, as 70% of the housing stock necessary to deliver on 2050 population growth projections has yet to be built^42,43^. The regional burden of radon-attributable lung cancer will only worsen if, as it is now, new housing stock continues to be built with an increasingly higher latent radon risk.

In terms of potential limitations, our sampling through citizen science enrollment was untargeted and, as we accepted all valid data from adult homeowners or renters, we consider selection biases to be minimized to the greatest possible extent. We acknowledge that our sampling methods may under-represent some in lower socioeconomic brackets, who may be less likely to prioritize radon test expenditures. As North American radon risks cannot yet be predicted accurately by property type, we consider it unlikely that our cohort is biased towards low or high radon-containing properties based on a willingness to participate. An essential future direction will be to consider the large-scale changes in residential occupancy (i.e. time spent at home versus at an office) elicited by the COVID-19 global pandemic, as well as general long term shifts towards working-from-home already occurring across workplaces. This information, weighted by age-of-exposure, should also serve as a future basis for long term radon-attributable lung cancer burden calculations.

In summary, we find that particle radiation exposure from North American residential radon is occurring at doses and dose rates of serious risk to humans, and which correspond to verified cancer-causing radon exposures in mammals. We also find that younger North Americans are being exposed inadvertently to greater radon gas levels due to intrinsic biases in occupancy of the newly-built residential environment. A major implication of this work is a strong rationale for public health authorities to refocus radon awareness and exposure prevention efforts to where it is likely to have the largest impact on reducing the future burden of radon-induced lung cancer, e.g. first-time homeowners and renters, especially those about to or just recently commencing parenthood. We also suggest that failure to address the underlying reasons for higher radon in newer residential properties will increase the likelihood of future radon-induced lung cancers occurring in younger and younger North Americans.

## Methods

### Consent, study design and participant eligibility

All methods were carried out in accordance with protocols approved by Research Ethics Boards (REBs), adhering to recommendations for research involving citizen science participants^31^. Study design, methods and data analysis were pre-approved by either the Conjoint Health Research Ethics Board, Research Services, University of Calgary (IDs = REB17-2239, REB19-1522, REB20-1729) or the Health Research Ethics Board of Alberta, Cancer Committee, and conferred (HREBA.CC-17-0246), as the ‘Evict Radon’ national study and sub-studies thereof. Records of informed consent were obtained in all cases. The survey region included all of Canada, with data in this study encompassing residential properties and occupants from: Yukon Territory, British Columbia, Alberta, Saskatchewan, Manitoba, Ontario, Quebec and Atlantic Provinces spread across > 250 distinct cities, towns and rural districts. The study operated using random, convenience recruitment (any who wanted to join). Recruitment methods included print media, public seminar, online (website and social media) and mass media messaging via organic (unpaid) TV/radio exposure in an untargeted manner. No data from any constituent part of this cohort were from known or pre-selected lung cancer cases. Homeowners and renters of any building type were equally eligible. Commercial offices or hospitality service buildings were not considered. Participants were permitted to withdraw at any time.

### Surveying

From 2015 to 020, Canadians purchased alpha track 90 + day radon detectors that they then deployed, returned for analysis, and later received their specific radon reading in a confidential manner. Non-profit study kits were inexpensive ($52), and were not considered to be a significant economic barrier to participation. Selection biases were considered, and socio-demographics adjusted for in analyses to the fullest extent. Following consent and placement of a radon test, all participants were invited to complete separate building metric, demographic and residential occupancy surveys using either Qualtrics or Hosted-in-Canada survey platforms. Of 18,971 invited participants from the ‘whole cohort’, all provided building metric data, and 3,518 in the ‘sub-study cohort’ additionally consented to provide and return detailed residence occupancy and/or demographic data. The relevant survey questions used to generate data for this particular study are outlined in Supplemental File 1.

### Radon testing

Radon tests were quality controlled by Canadian National Radon Proficiency Program (C-NRPP) certified professionals and distributed centrally by researchers. Care was taken to educate participants in the correct test deployment methods through print, video and direct communication with C-NRPP-certified professionals who adhere to Health Canada’s guidelines for long-term (90 + day) radon testing. Participants were advised to place devices on the lowest level of the building occupied for approximately four or more hours per day. As participants only place kits in areas and/or floors of the property where an occupant is spending a significant amount of time, we did not apply any correction factor(s) and chose to allow the radon readings to stand based on each individualized case of testing the air people are breathing regularly. For further rationale and methods concerning test placement, see^12^. Tests were deployed within residences for a minimum of 3 months if tests commenced between September to April, or for 6 months if tests commenced between May to August. Radon tests were RadTrak2 closed passive etched track detectors made from CR-39 plastic film inside antistatic holders enclosed in electrically conductive housing with filtered openings to permit gas diffusion, intended for long term (> 90 day) use with a typical linear range of 15 to 25,000 Bq/m^3^. To be read, CR-39 films are etched in 5.5 N NaOH at 70 °C for 15.5 min and scored using TrackEtch software at ISO17025 certified Radonova laboratories (Sweden, EU). Controls included (at no added cost to participants) precision and accuracy controls as described and reported in^12^. Readings throughout this study are in Bq/m^3^ rounded to the nearest whole number.

### Radiation exposure calculations

To convert Bq/m^3^ indoor air radon levels to human mSv radiation exposures (to lungs), the ICRP provides the following formula^44^:

$$\left( {6.7 \times 10^{ - 6} \;{\text{mSv }}per{\text{ Bq}}\;{\text{h}}/{\text{m}}^{3} } \right) \, \times \, \left( {\left[ {radon} \right]{\text{ Bq}}/{\text{m}}^{3} } \right) \, \times \, \left( {\left[ {time\; \, in\; \, residence\; \, per\; \, year} \right]{\text{ h}}/{\text{y}}} \right) \, = {\text{ mSv}}/{\text{y}}$$

Values for the amount of time spent in the primary residence per year for a typical adult (*“time in residence per year”)* were calculated from individually reported residential occupancy data from 1,128 participants, and cross-referenced with data from the *National Human Activity Pattern Study* (NHAPS)^32^. The NHAPS, which included responses from both Canadian and American respondents, estimated that 68.7% of life was spent inside a residence for the average adult. Of the total 8760 h per year, this equates to 6018 h/y and represents an average of all different employment statuses. Participants reported their data by season (Winter, Spring, Summer, Fall), with weekend/holiday versus workdays accounted for within the questionnaires (see Supplemental File 1). All response-derived “*time in residence per year”* outcomes were linked to individually-reported employment statuses and used to extrapolate the same values for all remaining participants (2,390) for which employment data was collected. Please note that for the purposes of participant responses in this survey, being enrolled in full or part time formal education (at a university, college, technical school, etc.) was considered to be a type of employment and grouped with those responses.

### Statistical analysis

Statistical analysis was carried out using Excel, Prism and R (4.0.2). ANOVAs were carried out to test differences between groups (e.g. year of construction, occupant age, mSv, etc.), with Bonferroni-Holm post-hoc testing carried out to characterize group differences for pairwise comparisons if the ANOVA reached significance.

### Data availability

The de-identified raw data sets generated by the current study are available to academic researchers at public institutions following reasonable request to the corresponding author, and will require a data transfer agreement. Data may not be used for private, commercial, or for-profit purposes for any reason.

## Supplementary Information

Supplementary Information.
