# Supplementary material for: Younger North Americans are exposed to more radon gas due to occupancy biases within the residential built environment
Source: Sci Rep. 2021 Mar 24;11:6724. doi: 10.1038/s41598-021-86096-3 (PMC7990966; doi:10.1038/s41598-021-86096-3)
Supplement: Supplementary file 1 — Supplementary Information. [file 41598_2021_86096_MOESM1_ESM.pdf]

SUPPLEMENTAL FILE 1

for

Younger North Americans are exposed to more radon gas due to occupancy biases within the residential built environment

Justin A. Simms[1*], Dustin D. Pearson[2*], Natasha L. Cholowsky[2], Jesse L. Irvine[2], Markus E. Nielsen[2], Weston R. Jacques[2], Joshua M. Taron[3], Cheryl E. Peters[4], Linda E. Carlson[5], Aaron A. Goodarzi[2**]

Author Affiliations:

[1]Faculty of Medicine, University of Saskatchewan, Saskatoon, Saskatchewan, Canada; [2]Robson DNA Science Centre, Departments of Biochemistry & Molecular Biology and Oncology, Charbonneau Cancer Institute, Cumming School of Medicine, University of Calgary, Calgary, Alberta, Canada; [3]School of Architecture and Landscape Planning, University of Calgary, Calgary, Alberta, Canada; [4]Departments of Cancer Epidemiology & Prevention Research and Community Health Sciences, Charbonneau Cancer Institute, Cumming School of Medicine, University of Calgary, Calgary, Alberta, Canada. [5]Division of Psychosocial Oncology, Department of Oncology Charbonneau Cancer Institute, Cumming School of Medicine, University of Calgary, Calgary, Alberta, Canada.

*These authors contributed equally

**Correspondence to Aaron Goodarzi (a.goodarzi@ucalgary.ca)

Participant Survey Questions Relevant to Simms, Pearson et al.

As part of approved studies REB17-2239, REB19-1522

1. Year of construction?

2. For the building tested, do you live in the property?

- Yes, full time
- Yes, part time
- No
- Prefer not to say or not sure

3. For the building tested, does any adult work in the property?

- Yes, full time
- Yes, part time
- No
- Prefer not to say or not sure

As part of approved study HREBA.CC-17-0246

1. What age were you at the time you decided to radon test your home (with our study)?

2. What was your biological sex at birth?
   a. Female
   b. Male
   c. Other______
   d. Prefer not to say

3. What was your employment status at the time you decided to obtain a radon test?
   a. Unemployed
   b. Full Time
   c. Part Time
   d. Retired
   e. Disability
   f. Prefer not to say

4. Do you or anyone else work from the home that was to be tested for radon?
   a. No
   b. Yes

5. How many children did you have at the time you decided to obtain a radon test?

6. How many people lived in the house at the time you decided to obtain a radon test?

7. Were there any minors (infants, toddlers, children, adolescents under age 18) who lived in or were regular visitors to the house at the time you decided to obtain a radon test?
   a. No
   b. Yes

As part of approved study REB20-1729

1. As of this date, approximately how many years have you lived in the property that you radon-tested via the Evict Radon study (regardless of whether or not you have moved)?

2. Do you still live in the residential property that you tested for radon with the Evict Radon study?

   a. Yes
   b. No

3. What is your age (as of this year)?

2. What was your status in terms of employment or enrollment in education?
(If answer "a" Go to Q3 / If answer "b" to "e" Go to Q4)

a. Full-time or part-time employed and/or enrolled in education**
   **These activities may take place at an office, school, or from your property. The next question(s) will ask you to tell us about where these are taking place.
b. Unemployed and/or not enrolled in education
c. Retired
d. On long-term leave (including maternity, disability, etc)
e. Prefer not to say

3. How many *hours per day* in each season did you typically spend:

	**INSIDE** your residence (tested by Evict Radon)	**INSIDE** a different residence	**INSIDE** a non-residential building (an office, store, business, hotel, school etc.)	**OUTDOORS** including time in a vehicle or transit	Total
Winter (Dec, Jan, Feb)	hours per day	hours per day	hours per day	hours per day	hours per day
Spring (Mar, Apr, May)	hours per day	hours per day	hours per day	hours per day	hours per day
Summer (Jun, Jul, Aug)	hours per day	hours per day	hours per day	hours per day	hours per day
Fall (Sep, Oct, Nov)	hours per day	hours per day	hours per day	hours per day	hours per day

4. On average how many *days per week* did you typically spend regularly attending work or school in each season (whether this was done in your residence or in another building)?

   a. days per week in "Winter" (Dec, Jan, Feb)
   b. days per week in "Spring" (Mar, Apr, May)
   c. days per week in "Summer" (Jun, Jul, Aug)
   d. days per week in "Fall" (Sep, Oct, Nov)

5. Approximately how many *hours per day* during a work or school day in each season did you typically spend:

	**INSIDE** your residence (tested by Evict Radon)	**INSIDE** a different residence	**INSIDE** a non-residential building (an office, store, business, hotel, school etc.)	**OUTDOORS** including time in a vehicle or transit	Total
Winter (Dec, Jan, Feb)	hours per day	hours per day	hours per day	hours per day	hours per day
Spring (Mar, Apr, May)	hours per day	hours per day	hours per day	hours per day	hours per day
Summer (Jun, Jul, Aug)	hours per day	hours per day	hours per day	hours per day	hours per day
Fall (Sep, Oct, Nov)	hours per day	hours per day	hours per day	hours per day	hours per day

6. Approximately how many *hours per day* during a weekend day or holiday in each season did you typically spend:

	**INSIDE** your residence (tested by Evict Radon)	**INSIDE** a different residence	**INSIDE** a non-residential building (an office, store, business, hotel, school etc.)	**OUTDOORS** including time in a vehicle or transit	Total
Winter (Dec, Jan, Feb)	hours per day	hours per day	hours per day	hours per day	hours per day
Spring (Mar, Apr, May)	hours per day	hours per day	hours per day	hours per day	hours per day
Summer (Jun, Jul, Aug)	hours per day	hours per day	hours per day	hours per day	hours per day
Fall (Sep, Oct, Nov)	hours per day	hours per day	hours per day	hours per day	hours per day

7. As of this date, and including yourself in the count, how many adults (18 or more years old) live regularly in your residence?

8. As of this date, how many minors (0 – 17 years old) live regularly in your residence?
